# Development of Droplet Digital PCR Assay for Detection of Seed-Borne *Burkholderia glumae* and *B. gladioli* Causing Bacterial Panicle Blight Disease of Rice

**DOI:** 10.3390/microorganisms10061223

**Published:** 2022-06-15

**Authors:** Jiannan Zhang, Jinyan Luo, Lei Chen, Temoor Ahmed, Saqer S. Alotaibi, Yanli Wang, Guochang Sun, Bin Li, Qianli An

**Affiliations:** 1 State Key Laboratory of Rice Biology, Ministry of Agriculture Key Lab of Molecular Biology of Crop Pathogens and Insects, Zhejiang Province Key Laboratory of Biology of Crop Pathogens and Insects, Institute of Biotechnology, College of Agricultural and Biotechnology, Zhejiang University, Hangzhou 310058, China; 21916084@zju.edu.cn (J.Z.); temoorahmed@zju.edu.cn (T.A.); libin0571@zju.edu.cn (B.L.); 2 Department of Plant Quarantine, Shanghai Extension and Service Center of Agriculture Technology, Shanghai 201103, China; toyanzi@126.com (J.L.); chenlei200524@163.com (L.C.); 3 Department of Biotechnology, College of Science, Taif University, P.O. Box 11099, Taif 21944, Saudi Arabia; saqer@tu.edu.sa; 4 State Key Laboratory for Managing Biotic and Chemical Threats to the Quality and Safety of Agro-Products, Zhejiang Academy of Agricultural Sciences, Hangzhou 310021, China; ylwang88@aliyun.com

**Keywords:** bacterial panicle blight of rice, *Burkholderia glumae*, *Burkholderia gladioli*, digital PCR

## Abstract

Bacterial panicle blight of rice or bacterial grain rot of rice is a worldwide rice disease. *Burkholderia glumae* and *B. gladioli* are the causal agents. The early and accurate detection of seed-borne *B. glumae* and *B. gladioli* is critical for domestic and international quarantine and effective control of the disease. Here, genomic analyses revealed that *B. gladioli* contains five phylogroups and the BG1 primer pair designed to target the 3’-end sequence of a gene encoding a Rhs family protein is specific to *B. glumae* and two phylogroups within *B. gladioli*. Using the BG1 primer pair, a 138-bp DNA fragment was amplified only from the tested panicle blight pathogens *B. glumae* and *B. gladioli*. An EvaGreen droplet digital PCR (dPCR) assay on detection and quantification of the two pathogens was developed from a SYBR Green real-time quantitative PCR (qPCR). The detection limits of the EvaGreen droplet dPCR on the two pathogens were identical at 2 × 10^3^ colony forming units (CFU)∙mL^−1^ from bacterial suspensions and 2 × 10^2^ CFU∙seed^−1^ from rice seeds. The EvaGreen droplet dPCR assay showed 10-fold detection sensitivity of the SYBR Green qPCR and could detect a single copy of the target gene in a 20-μL assay. Together, the SYBR Green qPCR assay allows for routine high-throughput detection of the panicle blight pathogens and the EvaGreen droplet dPCR assay provides a high-sensitive and high-accurate diagnostic method for quarantine of the pathogens.

## 1. Introduction

Bacterial panicle blight of rice or bacterial grain rot of rice is a worldwide rice disease and it is becoming a major threat to rice production because of global warming [1]. The symptoms are grain rot, sheath rot and seedling rot at different rice growth stages [2,3,4]. The management of the disease relies on the use of pathogen-free seeds, resistant rice cultivars and appropriate agricultural practices [1]. *Burkholderia glumae* was identified as the major causal agent, while *B. gladioli* was identified as the minor causal agent of the disease [1,3,5,6]. Both pathogens are seed-borne and can be transmitted over long distances via rice seeds [1,7,8]. Therefore, the early and accurate detection of *B. glumae* and *B. gladioli* in rice seeds is essential for effective control of the disease, epidemiological studies, and both domestic and international quarantine [1].

Detection and quantitation of specific nucleic acid sequences based on polymerase chain reaction (PCR) is fundamental to molecular diagnostic tests [9]. The first-generation PCR involves an end-point analysis of the presence or absence of the target sequence and a semi-quantification of the amplification product using gel or capillary electrophoresis. Real-time PCR or quantitative PCR (qPCR), the second-generation PCR technology, monitors the fluorescence emitted from the incorporation of a nucleic acid intercalating fluorescent dye (e.g., SYBR Green) or the hydrolysis of fluorescence-labeled probes (e.g., TaqMan probes) associated with the amplified target DNA from each amplification cycle and estimates the template abundance based on the fluorescent signal measured during the logarithmic amplification phase relative to an internal or external calibrator. The qPCR technique has become a mainstay of the diagnostic microbiology based on its relative quantification and high velocity, sensitivity, specificity and flexibility. Digital PCR (dPCR), the third-generation PCR technology, uses the same amplification reagents of qPCR but subdivides the reaction mixture into thousands of individual microscopic partitions to physically isolate the target molecules before their amplification. After end-point PCR, the starting concentration of template is determined by Poisson statistical analysis of the number of positive (digit 1) and negative (digit 0) reactions. The advantages of dPCR over qPCR include the absolute quantification without the need for a standard curve, a reduced template competition effect, bypassing PCR inhibitors in crude samples, and high detection sensitivity, leading to the increasing use of dPCR in the detection and precise quantification of low-level pathogens, rare genetic mutations, copy number variants, and relative gene expressions [9,10]. Partitioning of the PCR reaction can be created by different mechanisms, such as emulsified microdroplets suspended in oil, manufactured microwells, or microfluidic valving [10]. Recently, commercially available technology is able to subdivide a 20 μL homogeneous reaction mixture into about 20,000 highly uniform nanoliter-sized water-in-oil droplets and unify the partitioning, PCR amplification, and fluorescence detection systems into one platform, facilitating the droplet dPCR for routine use.

The transfer of probe-based qPCR assays to probe-based droplet dPCR assays has already provided successful detection and quantification of plant pathogenic bacteria [11,12,13,14,15,16,17]. Dye-based qPCR and dPCR assays are appealing due to reduced cost and complexity [18,19,20]. EvaGreen outperforms SYBR Green in qPCR and dPCR [19,20,21,22]. High-performance of dye-based qPCR and dPCR assays requires primers specific to the target sequences and without secondary structure formation and proper protocols [18].

SYBR Green qPCR assays have been established on detection and quantification of *B. glumae* [5,23]. The primers targeting the 16S–23S ribosomal RNA gene intergenic transcribed spacer sequence [5] generated non-specific amplification from *Burkholderia* and *Pseudomonas* [23]. Lee et al. also designed *B. glumae*-specific and *B. gladioli*-specific primers for qPCR [24]. This study aimed to develop droplet dPCR assays on the bacterial panicle blight pathogens *B. glumae* and *B. gladioli*. We tested two sets of *B. glumae*-specific primers BG1F/BG1R [23] and Bglu3F/Bglu3R [24] and found the BG1F/BG1R primers are specific to the tested *B. glumae* and *B. gladioli* strains. We thus developed an EvaGreen droplet dPCR assay on detection of seed-borne *B. glumae* and *B. gladioli* using BG1F/BG1R primers and analyzed the evolutionary divergence of *B. gladioli* based on genome sequences.

## 2. Materials and Methods

### 2.1. Bacteria Strains

Panicle blight pathogens *B. glumae* and *B. gladioli*, strains belonging to other *Burkholderia* species, other bacterial pathogens of rice used for PCR tests are presented in Table 1. Bacterial strains were grown in nutrient broth (10 g tryptone, 3 g beef extract, 2.5 g glucose, and 5 g NaCl per liter; pH 7.0) or on nutrient agar (nutrient broth with 15 g agar per liter) at 30 ℃.

### 2.2. Preparation of Bacterial Suspension and Rice Seeds Carrying Bacteria

*B. glumae* strain Os48 and *B. gladioli* strain Os50 grown to mid-exponential phase were suspended with sterile water to about 2 × 10^8^ colony-forming units (CFU)·mL^−1^. The bacterial suspensions were 10-fold serially diluted seven times. Each dilution was used as the template of bacterial suspension for qPCR and dPCR assays; the detection limits were determined at the cellular level.

Rice seeds of the cultivar Quanliangyou 1606 were surface-sterilized by immersion in 75% ethanol for 30 s, and in 3% sodium hypochlorite solution for 10 min, then washed six times with sterile water. Every 10 surface-sterilized seeds were immersed in 1 mL of the bacterial suspension (2 × 10^8^ CFU·mL^−1^) in a 1.5-mL centrifuge tube at 25 °C for 2 h. Seeds carrying bacteria were air-dried in sterile Petri dishes in a clean bench, and then immersed in 1 mL of sterile water in a 1.5-mL centrifuge tube. Seed-carrying bacteria were released by ultrasonic vibration at 53 KHz for 4 min. Released bacteria were grown on nutrient agar and counted as seed-carrying bacteria. Seed-carrying bacteria (about 2 × 10^5^ CFU·seed^−1^) released into water (about 2 × 10^6^ CFU·mL^−1^) were 10-fold serially diluted five times. Each dilution was used as the template of seed-carrying bacteria for qPCR and dPCR assays; the detection limits were determined at the cellular level.

### 2.3. Preparation of Bacterial Genomic DNA

Genomic DNA of bacterial strains grown in nutrient broth to late-exponential phase were extracted using a TIANamp Bacterial DNA Kit (Tiangen Biotech, Beijing, China). DNA quality and quantity were determined using a Qubit 3 Fluorometer (ThermoFisher Scientific, Waltham, MA, USA).

### 2.4. Genome Relatedness Analysis of Burkholderia gladioli

Whole genome sequences (WGS) of *B. gladioli* strains were obtained from the NCBI genome database (https://www.ncbi.nlm.nih.gov/genome/, accessed on 10 March 2022). Digital DDH (dDDH) values between pair genomes were calculated using the Genome-to-Genome Distance Calculator (http://ggdc.dsmz.de/distcalc2.php, accessed on 12 March 2022) with Formula (2); dDDH value of 79–80% was used for subspecies delimitation [35].

### 2.5. Phylogenomic Analysis

WGS of strains belonging to *B. glumae*, *B. gladioli*, or *B. plantarii* were annotated using the online platform Rapid Annotation using Subsystem Technology (RAST) version 2.0 (http://rast.nmpdr.org/, accessed on 12 March 2022) for pan-genome analysis. The phylogenomic tree was constructed based on the proteins encoded by their core genomes. *Burkholderia cepacia* ATCC 25416^T^ was selected as the outgroup. Orthologous clusters of proteins were analyzed and output by running the pan-genomes analysis pipeline (PGAP) [36]. Orthologs were determined by a BLAST E-value < 1e^−10^, sequence identity >50%, aligned sequence length coverage >50%, and score >40. The amino acid sequences from 2395 core proteins were concatenated and aligned using MAFFT version 5 [37]. The poorly aligned positions and excessively divergent regions were trimmed using GBlock 0.91b [38]. The resulting 739,894 amino acids were used to generate a maximum likelihood tree with the JTT + F + I + G4 model using the IQ-TREE version 2.1.2 [39]. The phylogenomic tree was displayed using the online tool iTOL version 6 [40].

### 2.6. Evaluation of Previously Designed B. glumae-Specific Primers

Specificity of two sets of previous *B. glumae*-specific primers BG1F/BG1R [23] and Bglu3F/Bglu3R [24] (Table 2) were validated by BLASTN, BLASTP, and alignment of the primer sequences against their target sequences in WGS and colony PCR against test strains (Table 1). DNA sequences were aligned using the MUSCLE program integrated in the MEGA5 software [41] and displayed using the BioEdit Sequence Alignment Editor version 7.2.5 [42].

Colony PCR was carried out as previously described [43]. A colony about 1 mm in diameter grown on nutrient agar was picked up with an autoclaved 10-μL pipette tip and transferred into 10 μL sterilized ultrapure water in a PCR tube. The bacterial suspension was heated in a P70F23P-G5 microwave oven (Galanz, Foshan, China) at full power for 3 min. After centrifugation, 1 μL of the bacterial lysate was used as the template for PCR. The primers were synthesized by Tsingke Biotechnology (Beijing, China). PCR was carried out in an S1000 thermal cycler (Bio-Rad Laboratories, Hercules, CA, USA). The PCR mixture (25 μL) contained 12.5 μL of 2 × Taq PCR Master Mix (Yeasen Biotechnology, Shanghai, China), 1 μL of each forward primer and reverse primer (10 μmol·L^−1^), and 9.5 μL of sterile ultrapure water. The PCR program was set as pre-denaturation at 94 °C for 5 min, 35 cycles of denaturation at 94 °C for 30 s, annealing at 63 °C (with BG1 primers) or 54 °C (with Bglu3 primers) for 30 s, and elongation at 72 °C for 10 s, and final elongation at 72 °C for 5 min. Ultrapure water without DNA was used as a negative control.

### 2.7. qPCR

The 10-fold serially diluted suspension of *B. glumae* strain Os48 and *B. gladioli* strain Os50 at 2 × 10^8^ to 2 × 10^2^ CFU·mL^−1^ and the 10-fold serially diluted seed-carrying bacteria at 2 × 10^5^ to 2 × 10^1^ CFU·seed^−1^ were used as the template for qPCR assays. Three replicates were prepared for the serial dilution. Ultrapure water was used as a negative control. qPCR was carried out in a CFX96 real-time PCR system (Bio-Rad Laboratories, Hercules, CA, USA).

SYBR Green qPCR mixture (20 μL) contained 10 μL of 2 × ChamQ SYBR Master Mix without ROX (Vazyme, Nanjing, China), 1 μL of template suspension, 0.4 μL of each BG1F primer and BG1R primer (10 μmol·L^−1^), and 8.2 μL of sterile ultrapure water. SYBR Green qPCR program was set as pre-denaturation at 95 °C for 30 s, 40 cycles of denaturation at 95 °C for 10 s and annealing/elongation at 63 °C for 30 s, and a melting curve of 65 to 95 °C with an increment of 0.5 °C. The SYBR Green qPCR assays were repeated three times.

### 2.8. dPCR

Droplet dPCR was carried out in an all-in-one Sniper DQ24 Digital PCR Platform (Sniper, Suzhou, China). Water-in-oil nano-droplets were generated by the vibrant injection technique developed by Sniper. Up to 16 dPCR mixtures were loaded into two 8-tube strips. Nano-droplets were generated from dPCR mixture and droplet generating oil onto four 4-well plates. About 23,000 nano-droplets (0.8 nL) were generated onto one well for one dPCR mixture (20 μL). Up to 16 samples can be tested in one run.

A thermal gradient ranging from 58 to 64 °C was tested to determine the optimal annealing temperature. Bacterial genomic DNA was adjusted to 1.5 ng·μL^−1^ and used as template for dPCR. Ultrapure water was used as a negative control.

The 10-fold serially diluted templates of bacterial suspension and seed-carrying bacteria for dPCR at the optimal annealing temperature were prepared as described for qPCR. The EvaGreen dPCR mixture (20 μL) contained 10 μL of 2 × HQ dPCR EvaGreen Master Mix (Sniper, Suzhou, China), 1 μL of template suspension, 0.5 μL of each BG1F primer and BG1R primer (10 μmol·L^−1^), and 8 μL of sterile ultrapure water. EvaGreen dPCR program was set as droplet generation at 60 °C for 5 min, pre-denaturation at 95 °C for 5 min, 40 cycles of denaturation at 95 °C for 30 s and annealing/elongation at 60 °C for 45 s. Ultrapure water was used as a negative control. Every EvaGreen dPCR assay was repeated three times.

After amplification, the fluorescent signals of the droplets on the 4-well plates were detected by the build-in fluorescence detector (Sniper). Data acquisition and analysis were performed by SightPro software (Sniper). For each well, a threshold was automatically and manually set just above the amplitude value of the cloud corresponding to the negative droplets, which was also considered as the background. A result was considered positive if at least two positive droplets were detected. The concentration (*C*) of the target DNA (copy·well^−1^) was provided using the formula: *C* = −In [1 − *P*/(*P* + *N*)] × 1/*V*, where *P* is the positive droplet number, *N* is the negative droplet number, and *V* is the mean volume in μL of one droplet (0.8 × 10^−3^ μL). Because the target gene is present in a single copy in the *B. glumae* and *B. galdioli* genomes, the results can be converted into copy·μL^−1^ in the initial samples by multiplying *C* with the total volume of reaction mixture (20 μL) and then dividing by the volume of the template added to the reaction mixture (1 μL).

## 3. Results and Discussion

### 3.1. BG1 Primers Are Specific to B. glumae and Two Phylogroups within B. gladioli

**Figure 1:**
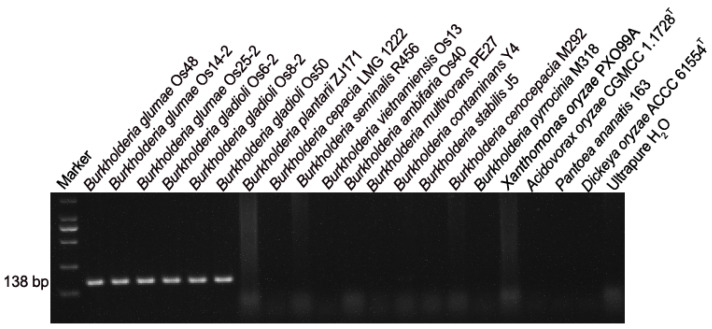
Agarose gel electrophoresis showing PCR amplification of 138-bp DNA fragments with BG1 primers from *Burkholderia glumae* and *B. gladioli* strains.

BG1 primers target to a 138-bp DNA sequence encoding the C-terminal region of a Rhs family protein, which is also annotated as RHS repeat-associated core domain protein and DUF6531 domain-containing protein. Kim et al. showed that PCR amplification with BG1 primers was positive only on *B. glumae* strains but not on the tested *B. gladioli* type strain LMG 2216^T^ [23]. However, here, PCR amplification was positive not only on the three *B. glumae* strains but also on the three *B. gladioli* strains (Figure 1). BLASTN search of the 138-bp target sequence against the NCBI non-redundant nucleotide collection revealed that *B. gladioli* strain FDAARGOS_389 chromosome 1 (accession no. CP023522.1) and *B. gladioli* strain FDAARGOS_951 plasmid 1 (accession no. CP065597.1) contained sequences highly similar to the target sequences in *B. glumae*. Moreover, BLASTP search of the target Rhs family protein (ID AJY63123.1) against the NCBI non-redundant protein sequences revealed that some *B. gladioli* strains contained the target Rhs family protein.

Bglu3 primers target to a 174-bp DNA sequence encoding a hypothetical protein. Lee et al. showed that PCR amplification with Bglu3 primers was positive only on *B. glumae* strains but not on the six tested *B. gladioli* strains [24]. Here, PCR amplification was also positive only on the three *B. glumae* strains but not on the three *B. gladioli* strains (Data not shown). However, BLASTP search of the target hypothetical protein containing 61 amino acids (ID WP_017432160.1) against the NCBI non-redundant protein sequences revealed that the target hypothetical protein is similar (identity 70.49%) to the C-terminal region of a DUF6531 domain-containing protein containing 1579 amino acids (such as ID WP_186165364.1) in some *B. gladioli* strains.

**Figure 2:**
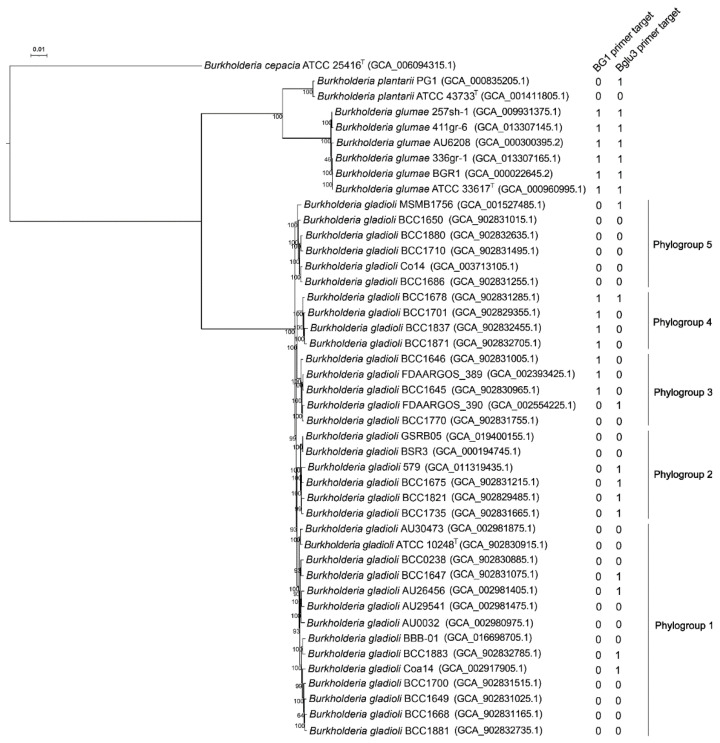
Phylogenomic tree based on 739,894 amino acids from concatenated 2395 core proteins of strains belonging to *Burkholderia gladioli*, *B. glumae*, *B. plantarii*, and the outgroup strain *B. cepacia* ATCC 25416^T^. Genome assembly identifier numbers are shown in brackets after the strain name. Presence (1) and absence (0) of the BG1-primer-targeting sequence and the Bglu3-primer-targeting sequence are listed after the genome assembly identifier. *Burkholderia gladioli* forms five phylogenetic groups (Phylogroup 1–5). Bootstrap values of 1000 tests are shown at the nodes. The scale bar indicates 0.01 substitutions per site.

*Burkholderia gladioli* occupies divergent ecological niches and is in association with a broad spectrum of hosts and diseases in humans, animals and plants. Recent phylogenomic analyses have shown that *B. gladioli* contains five clades [44,45,46]. To find the distribution of the BG1-primer-targeting protein and the Bglu3-primer-targeting protein in *B. gladioli*, we did a phylogenomic analysis on closely related *B. gladioli*, *B. glumae*, and *B. plantarii*. The phylogenomic analysis showed that *B. glumae* is more closely related to *B. plantarii* than *B. gladioli* and that *B. gladioli* contains five phylogroups (Figure 2). *B. gladioli* phylogroup 1 contains the type strain ATCC 10248^T^ (= LMG 2216^T^) (Figure 2). Genome relatedness analysis showed that phylogroup 1 shares dDDH similarities with other four phylogroups slightly above the dDDH threshold (79–80%) for subspecies delimitation [35], while phylogroup 3, 4, and 5 share dDDH similarities slightly below the dDDH threshold or at the border of the threshold for subspecies delimitation (Table S1). It is likely that the five phylogroups nearly differentiated into five subspecies.

The BG1-primer-targeting sequences are present not only in all (15 of 15) *B. glumae* strains but also in all (10 of 10) strains within the *B. gladioli* phylogroup 4 and most (63 of 88) strains within the *B. gladioli* phylogroup 3. Although the strains within the phylogroup 3 and 4, whose WGS had been released into the NCBI genome database, were isolated from humans or environments, the three tested *B. gladioli* strains in this study likely belong to the phylogroup 3 and 4. Moreover, the panicle blight pathogens belonging to *B. gladioli* are not restricted to the phylogroup 3 and 4 because the virulent strain BSR3 isolated from diseased rice sheath belongs to the phylogroup 2 (Figure 2).

Here, the *B. gladioli* phylogroups 1, 2, 3, 4, and 5 correspond to the previous *B. gladioli* Clade 3, 1B, 2, 1C, and 1A [44,45], respectively. Phylogroups 2, 4, and 5 were previously named Clade 1A, 1B, and 1C because they all contained the bongkrekic acid biosynthetic gene cluster [44,45]. The gladiolin biosynthetic gene cluster is restricted to clade 3 (=phylogroup 1). *B. gladioli* pv. *allicola* associated with onion soft-rot disease is restricted to previous clade 2 (=phylogroup 3).

The Bglu3-primer-targeting sequences are present in all *B. glumae* strains, some strains of each *B. gladioli* phylogroup, and *B. plantarii* strain PG1 (Figure 2).

**Figure 3:**
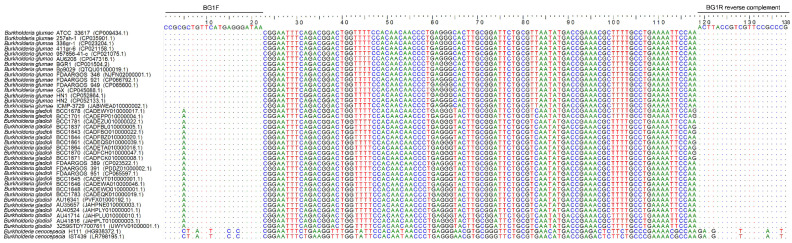
DNA alignment of BG1F primer, BG1R primer reverse complement, and their 138-bp target sequences in the *Rhs* family gene. The accession numbers of the target sequences in NCBI database are shown in brackets after the strains names. Dots (∙) indicate identical nucleotides aligned at the same site as the BG1 primers; letters A, T, G, and C indicate different nucleotides aligned at the same site.

DNA alignment showed that the BG1F primer sequence is identical to the target region in the 138-bp target sequences of all *B. glumae* genomes and is different from the target region in *B. gladioli* genomes at the 5th position of the 20 nucleotides (Figure 3); the BG1R primer sequence is identical to the target region in all *B. glumae* genomes and the *B. gladioli* genomes containing the 138-bp target sequences (Figure 3). The mismatch of one nucleotide at the 5th position of the 20 nucleotides of BG1F may not substantially reduce the amplification efficiency. Consistent with the PCR amplification result (Figure 1), BG1F/BG1R primers are specific to *B. glumae* and the phylogroup 3 and 4 within *B. gladioli*.

**Figure 4:**
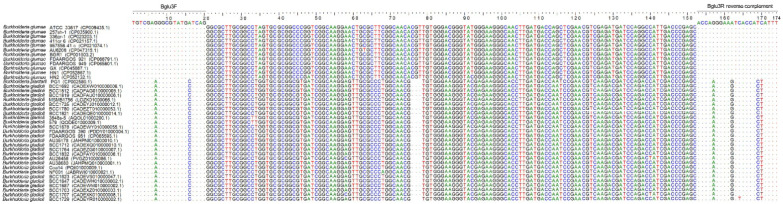
DNA alignment of Bglu3F primer, Bglu3R primer reverse complement, and their target sequences in *Burkholderia glumae* and *B. gladioli* genomes. The accession numbers of the target sequences in NCBI database are shown in brackets after the strains names. Dots (∙) indicate identical nucleotides aligned at the same site as the Bglu3 primers; letters A, T, G, and C indicate different nucleotides aligned at the same site.

DNA alignment showed that the Bglu3F primer sequence is identical to the target region in the 174-bp target sequences of all *B. glumae* genomes and is different from the target region in *B. gladioli* genomes at the 7th and 16th positions of the 20 nucleotides (Figure 4); the Bglu3R primer sequence is identical to the target region in all *B. glumae* genomes and is different from the target region in *B. gladioli* genomes at the 5th, 6th, 13th, and 18th positions from the 5′-end of the 22 nucleotides (Figure 4). The mismatch of the two nucleotides in the Bglu3F region and particularly the four nucleotides in the Bglu3R region may substantially reduce the amplification efficiency. The mismatch of the Bglu3 primers to the target sequences in *B. gladioli* genomes or the absence of the target sequences in the genomes of the three tested *B. gladioli* strains are likely the reason for the negative result of the PCR amplification with the Bglu3 primers from the three tested *B. gladioli* strains.

Because the BG1 primers are specific to *B. glumae* and the tested *B. gladioli* strains, the BG1 primers were used for further qPCR and dPCR assays on detection of the rice panicle blight pathogens *B. glumae* and *B. gladioli*.

### 3.2. Detection of B. glumae and B. gladioli Using SYBR Green qPCR

**Figure 5:**
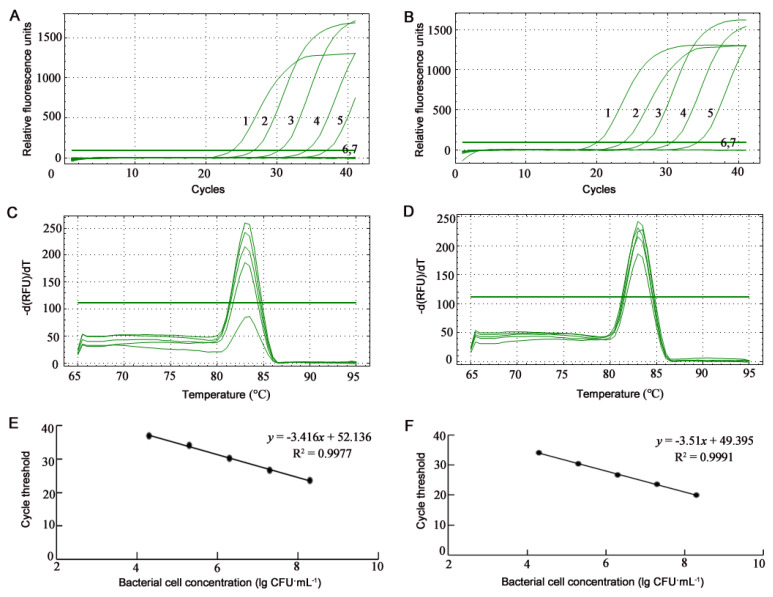
Profiles of SYBR Green real-time quantitative PCR (qPCR) assays on bacterial suspensions of *Burkholderia glumae* strain Os48 (**A**,**C**,**E**) and *B. gladioli* strain Os50 (**B**,**D**,**F**). (**A**,**B**) Amplification curves of SYBR Green qPCR assays. Numbers 1, 2, 3, 4, 5, and 6 indicate the 1-μL templates from bacterial suspensions at the concentration of 2 × 10^8^, 2 × 10^7^, 2 × 10^6^, 2 × 10^5^, 2 × 10^4^, and 2 × 10^3^ CFU·mL^−1^, respectively; 7 is ultrapure water without template. (**C**,**D**) Melting curves of SYBR Green qPCR assays show the single specific peak at Tm of 83 °C. (**E**,**F**) Standard curves of SYBR Green qPCR assays show a linear response between bacterial cell concentration (lg CFU·mL^−1^) and cycle threshold.

**Figure 6:**
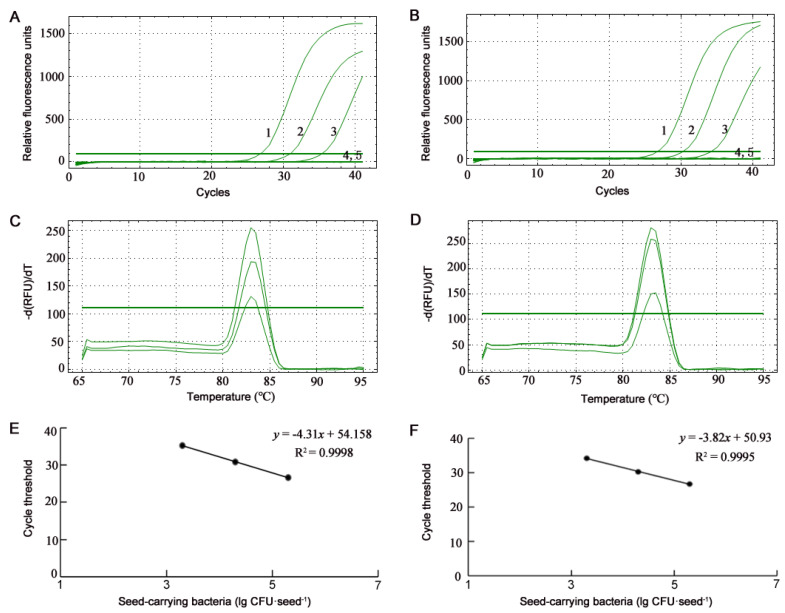
Profiles of SYBR Green real-time quantitative PCR (qPCR) assays on seed-carrying bacterial strains *Burkholderia glumae* Os48 (**A**,**C**,**E**) and *B. gladioli* Os50 (**B**,**D**,**F**). (**A**,**B**) Amplification curves of SYBR Green qPCR assays. Numbers 1, 2, 3, and 4 indicate the 1-μL templates from seed-carrying bacteria at the concentration of 2 × 10^5^, 2 × 10^4^, 2 × 10^3^, and 2 × 10^2^ CFU·seed^−1^, respectively; 5 is the template from ultrapure water-treated seeds. (**C**,**D**) Melting curves of SYBR Green qPCR assays show the single specific peak at Tm of 83 °C. (**E**,**F**) Standard curves of SYBR Green qPCR assays show a linear response between bacterial cell concentration (lg CFU·seed^−1^) and cycle threshold.

The melting curves of the SYBR Green qPCR amplification with the BG1 primers from cells of *B. glumae* Os48 and *B. gladioli* Os50 repeatedly showed the single specific peak at Tm of 83 °C (Figure 5C,D and Figure 6C,D) and no primer-dimer formation. The standard curves of the SYBR Green qPCR were constructed by plotting the mean cycle threshold (C_T_) (*n* = 3) versus logarithmic concentrations of bacterial cells in suspensions ranging from 2 × 10^8^ to 2 × 10^4^ CFU·mL^−1^ (Figure 5E,F) and in artificially inoculated seeds ranging from 2 × 10^5^ to 2 × 10^3^ CFU·seed^−1^ (Figure 6E,F) and displayed linear responses. The detection limit of the SYBR Green qPCR on *B. glumae* and *B. gladioli* cells were identical; both were 2 × 10^4^ CFU·mL^−1^ from bacterial suspensions and 2 × 10^3^ CFU·seed^−1^ from artificially inoculated seeds. Because 2 × 10^3^ CFU·seed^−1^ of artificially inoculated seeds was equal to the suspension at 2 × 10^4^ CFU·mL^−1^ released from the 10 artificially inoculated seeds, the detection limits from bacterial suspensions and artificially inoculated seeds were also identical. Because the 20-μL qPCR assay contained 1 μL template suspension and the target gene is present in a single copy in the *B. glumae* and *B. galdioli* genomes, the detection limits of the qPCR were 20 cells and 20 copies of the target gene in the 20-μL reaction. If the 20-μL qPCR assay contained up to 9.2 μL of the template suspension, the qPCR detection limit can reach the level of 2 × 10^2^ CFU·seed^−1^ from artificially inoculated seeds.

### 3.3. Detection of B. glumae and B. gladioli Using Droplet EvaGreen dPCR

**Figure 7:**
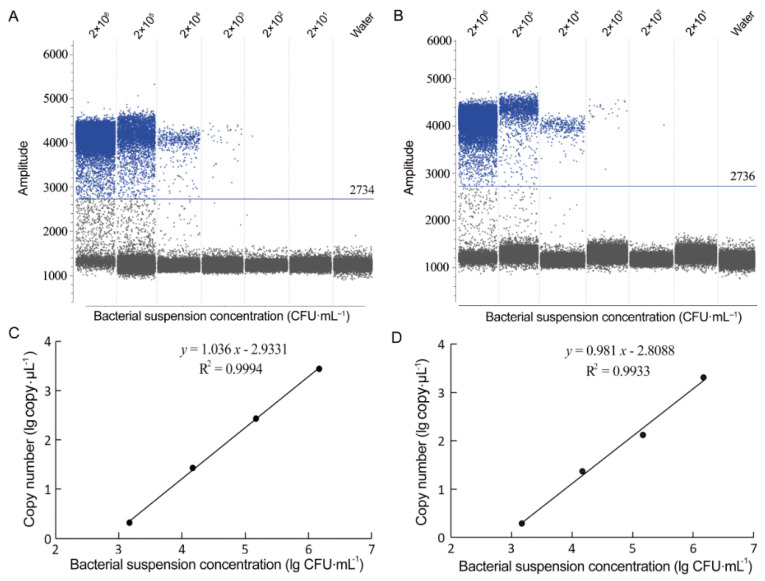
Profiles of EvaGreen droplet digital PCR (dPCR) assays on bacterial suspensions of *Burkholderia glumae* strain Os48 (**A**,**C**) and *B. gladioli* strain Os50 (**B**,**D**). (**A**,**B**) Fluorescence amplitude 1-D plot of EvaGreen droplet dPCR assays on bacterial suspensions at the 10-fold diluted concentrations from 2 × 10^6^ to 2 × 10 CFU·mL^−1^; ultrapure water was used as the no template control. Blue line shows the threshold separating negative droplets from positive droplets; blue dots indicate positive droplets of amplification; grey dots indicate negative droplets of no amplification. One positive droplet (<2) detected at the concentration of 2 × 10^2^ CFU·mL^−1^ was not considered as positive. (**C**,**D**) Regression curves of EvaGreen droplet dPCR show a linear response between bacterial cell concentration (lg CFU·mL^−1^) and copy number of detected target DNA.

**Figure 8:**
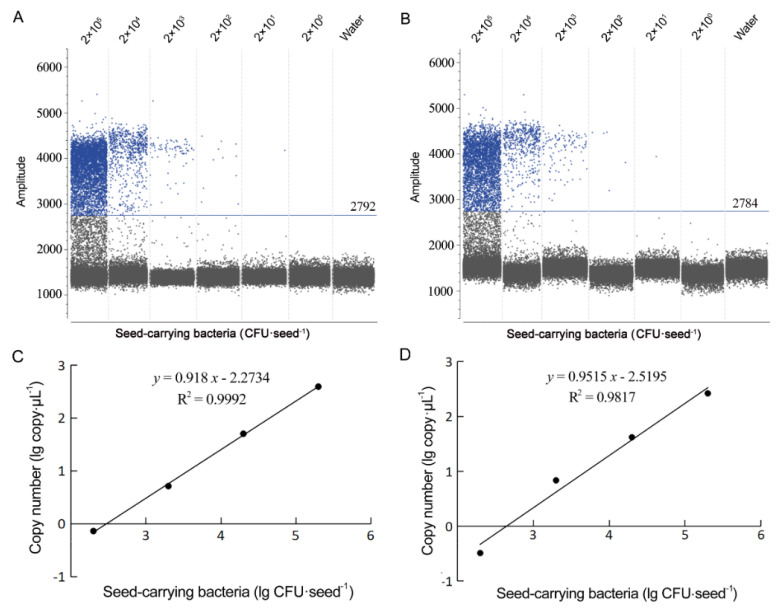
Profiles of EvaGreen droplet digital PCR (dPCR) assays on seed-carrying bacterial strains *Burkholderia glumae* Os48 (**A**,**C**) and *B. gladioli* Os50 (**B**,**D**). (**A**,**B**) Fluorescence amplitude 1-D plot of EvaGreen droplet dPCR assays on seed-carrying bacteria at the 10-fold diluted concentrations from 2 × 10^5^ to 2 × 10^0^ CFU·seed^−1^; aqueous solutions from mock inoculation with ultrapure water were used as the no template control. Blue line shows the threshold separating negative droplets from positive droplets; blue dots indicate positive droplets of amplification; grey dots indicate negative droplets of no amplification. One positive droplet (<2) detected at the concentration of 2 × 10^1^ CFU·seed^−1^ was not considered as positive. (**C**,**D**) Regression curves of EvaGreen droplet dPCR show a linear response between bacterial cell concentration (lg CFU·seed^−1^) and copy number of detected target DNA.

The optimal annealing temperature for EvaGreen-based droplet dPCR using BG1 primers was 60 °C, because at this temperature, positive droplets showed the highest fluorescence amplitude and a clear separation from the negative droplets (Data not shown). In the EvaGreen droplet dPCR, optimal quantification on both *B. glumae* and *B. gladioli* ranged from 2 × 10^3^ to 2 × 10^6^ CFU·mL^–1^ for bacterial suspension (Figure 7) and from 2 × 10^2^ to 2 × 10^5^ CFU·seed^–1^ for seed-carrying bacteria (Figure 8). Bacterial cell concentrations were positively correlated with droplet dPCR copy numbers (Figure 7C,D and Figure 8C,D). The detection limits of the EvaGreen droplet dPCR on *B. glumae* and *B. gladioli* were identical; both were 2 × 10^3^ CFU·mL^−1^ from bacterial suspensions and 2 × 10^2^ CFU·seed^−1^ from artificially inoculated seeds (Figure 7 and Figure 8). Because 2 × 10^2^ CFU·seed^−1^ of artificially inoculated seeds was equal to the suspension at 2 × 10^3^ CFU·mL^−1^ released from the 10 artificially inoculated seeds, the detection limits from bacterial suspensions and artificially inoculated seeds were also identical. The detection limit of the dPCR assays with 1 μL template suspension was 2 cells and 2 copies of the target gene in the 20-μL reaction. If the 20-μL dPCR assays contained up to 9 μL of the template suspension, the dPCR test can detect *B. glumae* and *B. gladioli* positively from seeds containing the pathogen cells about 20 CFU·seed^−1^.

The detection limit of the EvaGreen droplet dPCR was one-tenth of that of the SYBR Green qPCR; the detection sensitivity of the EvaGreen droplet dPCR was 10-fold of that of the SYBR Green qPCR and reached the level of single-copy, practically the highest sensitivity for detection of nucleic acid sequences.

The all-in-one Sniper DQ24 Digital PCR Platform can process the droplet dPCR automatically. A fluorescent dye-based droplet dPCR assay may take almost the same time as a qPCR assay, replacing the time for melting curve in qPCR by droplet generation in dPCR. The droplet dPCR needs droplet-generating oil and consumables including disposable droplet-generating needles/boxes, 4-well plates and plate sealing covers and thus incurs extra costs. In qPCR, each sample needs to be run in triplicate and the generation of standard curves incurs extra costs, narrowing the cost gap between droplet dPCR and qPCR. Another disadvantage of the droplet dPCR assay is the lower 16-well throughput compared with the 96-well qPCR assays.

## 4. Conclusions

We developed the EvaGreen droplet dPCR assay on detection and quantification of rice-seed-borne panicle blight pathogens of *B. glumae* and *B. gladioli* using the previous *B. glumae*-specific BG1 primers [23]. BG1 primers specific to the target sequences and without secondary structure formation are the key for supporting the high performance of the SYBR Green qPCR and the EvaGreen droplet dPCR on detection of the target *B. glumae* and *B. galdioli*. The EvaGreen droplet dPCR showed 10-fold detection sensitivity of the SYBR Green qPCR and could detect single copy of the target gene in a 20-μL assay. While the SYBR Green qPCR assay allows for routine high-throughput detection of the panicle blight pathogens, the EvaGreen droplet dPCR assay provides a highly accurate method of developing accurate standard curves for the qPCR method and a highly sensitive method for the detection of the panicle blight pathogens for both domestic and international quarantine.

We extended the specificity of the BG1 primers from *B. glumae* to two of the five phylogroups within *B. gladioli*. The five *B. gladioli* phylogroups nearly differentiated into five subspecies. The BG1 primers are likely not suitable for detection of all *B. gladioli* panicle blight pathogens. Duplex qPCR and dPCR assays using both the *B. glumae*-specific Bglu3 primers and the *B. gladioli*-specific Bgla9 primers [24] to distinguish *B. glumae* from *B. gladioli* and detect all panicle blight pathogens are better approaches for future use.

Whether the *B. gladioli* panicle blight pathogens are restricted to certain phylogroups is not clear. The specific genetic factors linked to the separation of *B. gladioli* phylogroups are also not clear. Further genomic analyses of diverse *B. glumae* and *B. gladioli* including worldwide panicle blight pathogens will lead to a better understanding of the evolution and virulence of the panicle blight pathogens.

## Figures and Tables

**Table 1:** Bacterial strains used for PCR.

Bacterial Strains	Isolation Source	Reference
*Burkholderia glumae* Os48	Rice	[25]
*Burkholderia glumae* Os14-2	Rice	This study
*Burkholderia glumae* Os25-2	Rice	This study
*Burkholderia gladioli* Os6-2	Rice	This study
*Burkholderia gladioli* Os8-2	Rice	This study
*Burkholderia gladioli* Os50	Rice	[25]
*Burkholderia cepacia* LMG 1222^T^	Onion	[26]
*Burkholderia plantarii* ZJ171	Rice paddy	[27]
*Burkholderia cenocepacia* M292	Maize rhizosphere	[28]
*Burkholderia pyrrocinia* M318	Maize rhizosphere	[28]
*Burkholderia vietnamiensis* Os13	Rice	[25]
*Burkholderia ambifaria* Os40	Rice	[25]
*Burkholderia seminalis* R456	Rice rhizosphere	[29]
*Burkholderia multivorans* PE27	Lake water	[30]
*Burkholderia stabilis* J5	Lake water	[30]
*Burkholderia contaminans* Y4	Human sputum	[31]
*Xanthomonas oryzae* pv. *oryzae* PXO99A	Rice	[32]
*Acidovorax oryzae* CGMCC 1.1728^T^	Rice	[33]
*Dickeya oryzae* ACCC 61554^T^	Rice	[34]
*Pantoea ananatis* F163	Rice	This study

**Table 2:** PCR-primers tested for detection of *Burkholderia glumae* and *B. gladioli*.

Primer Name	Primer Sequence (5′–3′)	Target Sequence Length (bp)	Target Protein
BG1F	CCGCGCTGTTCATGAGGGATAA	138	Rhs family protein
BG1R	CGGGCGGAACGACGGTAAGT		
Bglu3F	TGTCGAGGGCGTATGATCAG	174	Hypothetical protein
Bglu3R	AAATGATGGTGATTTCCCTGGT		

## Data Availability

Not applicable.

## Supplementary Materials

The following are available online at https://www.mdpi.com/article/10.3390/microorganisms10061223/s1. Table S1: Digital DNA-DNA hybridization (dDDH) values between genome pairs of strains belonging to *Burkholderia gladioli*.
